# Changes in composition and abundance of functional groups of arctic fungi in response to long-term summer warming

**DOI:** 10.1098/rsbl.2016.0503

**Published:** 2016-11

**Authors:** József Geml, Tatiana A. Semenova, Luis N. Morgado, Jeffrey M. Welker

**Affiliations:** 1 Naturalis Biodiversity Center, PO Box 9517, 2300 RA Leiden, The Netherlands; 2 Faculty of Science, Leiden University, PO Box 9502, 2300 RA Leiden, The Netherlands; 3 Department of Biological Sciences, University of Alaska Anchorage, Anchorage, AK 99508, USA

**Keywords:** climate change, fungal ecology, metabarcoding, tundra

## Abstract

We characterized fungal communities in dry and moist tundra and investigated the effect of long-term experimental summer warming on three aspects of functional groups of arctic fungi: richness, community composition and species abundance. Warming had profound effects on community composition, abundance, and, to a lesser extent, on richness of fungal functional groups. In addition, our data show that even within functional groups, the direction and extent of response to warming tend to be species-specific and we recommend that studies on fungal communities and their roles in nutrient cycling take into account species-level responses.

## 1. Introduction

The arctic tundra is considered a maritime biome, as approximately 80% of non-alpine tundra is located within 100 km of a coastline [1]. As a result of the retreating sea ice, arctic land surface temperatures are increasing, causing major changes in terrestrial ecosystems [2,3]. In response to warming temperatures, shifts in land surface vegetation and ecosystem C cycling have already been observed in terrestrial arctic ecosystems [3,4]. However, the responses of belowground communities, such as soil microbes, have been less certain [5].

Fungi are key to the functioning of terrestrial arctic ecosystems as symbionts (e.g. mycorrhizae, endophytes and lichens) and decomposers. Given their intimate relationships with plants in a wide range of symbioses, fungi are expected to play an important role in arctic vegetation change [6]. In this study, we compared fungal communities across plots with ambient and experimentally increased summer air and near-surface soil temperature to reveal (i) how community composition and abundance of functional groups of fungi change in response to long-term increase in summer temperature and (ii) whether these responses are similar in dry and moist tundra.

## 2. Material and methods

### (a) Data generation

The study was conducted at the Toolik Field Station in Alaska, USA, where the main vegetation types are dry acidic heath and moist acidic tussock tundra [7,8]. Open top chambers (OTCs), with 1 m^2^ area and 0.4 m height, were established in 1994 in both tundra types to increase summer air and upper soil temperature by approximately 2°C, leading to shifts in edaphic factors and vegetation [7–9].

We sampled 100 soil cores across 20 plots: five replicate plots in the OTCs and control plots in each tundra type, with five randomly collected soil cores of 2 cm diameter and 20 cm depth per plot which were mixed and lyophilized. We extracted DNA using the Macherey–Nagel NucleoSpin-Soil kit. PCR and sequencing of the ITS2 (internal transcribed spacer 2) rDNA were done as described earlier [10–12]. We generated 4 047 811 reads using Ion 318™ Chip (http://dx.doi.org/10.5061/dryad.2fc32).

### (b) Bioinformatics

Primers and adapters were removed and poor-quality ends were trimmed off using 0.02 error probability limit in Geneious Pro 5.6.1. Sequences were truncated to 200 bp and sequences with expected error more than 1 were discarded using usearch v. 8.0 [13]. The remaining 1 632 682 sequences were collapsed into unique sequence types on a per-sample basis while preserving read counts. After discarding singletons, 1 092 238 high-quality sequences were grouped into 4069 operational taxonomic units (OTUs) with usearch at 97% sequence similarity, while excluding 9026 (0.3%) chimeric sequences. We identified 3501 OTUs based on the UNITE fungal database, discarding OTUs with less than 70% similarity to any fungal sequence.

We assigned ecological functions to 1655 OTUs based on taxonomic identities of the matching reference sequences and following functional classifications in [14]: arbuscular mycorrhizal (5 OTUs), animal parasitic (18), ectomycorrhizal (417), lichenicolous (9), lichenized (156), mycoparasitic (39), plant pathogenic (134) and saprotrophic (877) fungi. Because of low richness, arbuscular mycorrhizal fungi were excluded, while animal- and mycoparasites were combined, as were lichens and lichenicolous fungi.

### (c) Statistical analyses

For each functional group, OTU richness (S), Shannon's and Simpson's diversity indices were calculated in pc-ord v. 6.0 [15] based on abundance matrix and were compared using two-way ANOVA to test for effects of warming, tundra type and their interaction. We visualized changes in community composition of functional groups with non-metric multidimensional scaling (NMDS) based on presence–absence data with Bray–Curtis distance and 500 iterations in pc-ord. We tested for statistical difference in fungal community composition among tundra types and treatments using multi-response permutation procedure (MRPP). We prepared Venn diagrams of the five major functional groups to show the number of shared OTUs among tundra and treatment types using venny [16].

We assessed the effect of warming on abundance on a per-OTU basis by comparing DNA sequence counts (Hedges' *D*) and calculating the mean effect size with 95% confidence intervals using metawin v. 2.0 [17]. Using sequence read counts as a proxy for abundance (biomass) is constrained owing to interspecific differences in copy number and length of ITS [18]. However, for individual OTUs, changes in sequence counts can indicate relative changes in abundance (biomass) [18]. We compared per-OTU mean read counts across the control and warmed plots to calculate effect sizes with variance and calculated mean effect size with 95% confidence interval for each functional group. This approach allowed us to depict the variation in responses of individual OTUs to warming and evaluate the overall responses of functional groups.

## 3. Results

### (a) Diversity measures

Tundra type had the strongest effect on lichens, where all diversity measures were significantly higher in the dry tundra (table 1). Similarly, in the animal- and mycoparasitic fungi, both Shannon's and Simpson's diversity indices were higher in the dry tundra, even though differences in richness were insignificant. Warming only affected richness in ectomycorrhizal fungi, with strong decrease in the moist tundra, although Shannon's and Simpson's diversity indices were not significantly affected. A similar, but somewhat weaker trend was seen in lichens. Shannon's diversity decreased in saprotrophic fungi, even though neither richness nor Simpson's diversity was strongly affected. The interaction of warming and tundra type showed significant decrease in richness in ectomycorrhizal and saprotrophic fungi, and only in saprotrophs regarding Shannon's and Simpson's diversity.

**Table 1.** The results (*p*-values) of two-way ANOVA on OTU richness, Shannon's and Simpson's diversity indices calculated for functional groups of fungi. Significant (*p* < 0.05) effects are indicated in italics. Abbreviations: ECM, ectomycorrhizal fungi; AP, animal parasites; MP, mycoparasites; LIC, lichens and lichenicolous fungi; PP, plant pathogens; SAP, saprotrophs.

index	effects	ECM	AP+MP	LIC	PP	SAP
richness (*S*)	treatment (warming)	*0.0168*	0.3932	0.069	0.6171	0.2476
	tundra type (dry versus moist)	0.2692	0.604	*<0.0001*	0.531	0.5854
	treatment × tundra type	*0.0176*	1	0.5795	0.4854	*0.0477*
Shannon's diversity (*H*)	treatment (warming)	0.2623	0.0881	0.0782	0.494	*0.0324*
	tundra type (dry versus moist)	0.1237	*0.0309*	*<0.0001*	*0.036*	0.2213
	treatment × tundra type	0.8647	0.7132	0.844	0.4612	*0.0023*
Simpson's diversity (*D*)	treatment (warming)	0.373	0.0541	0.2935	0.6529	1
	tundra type (dry versus moist)	0.1313	*0.0368*	*0.0001*	0.0693	1
	treatment × tundra type	1	1	0.5028	0.6529	*0.001*

### (b) Community composition

**Figure 1.**
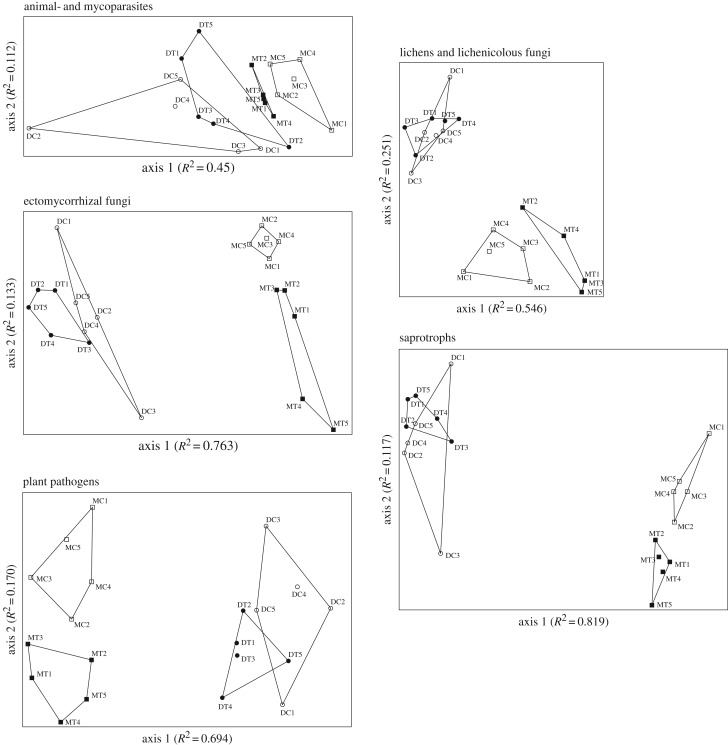
Non-metric multidimensional scaling (NMDS) ordination plots for functional groups of arctic fungal communities in the warmed and control plots in the dry and moist tundra types based on presence–absence. M = moist tundra, D = dry tundra, C = control, T = warming.

NMDS analyses resulted in two-dimensional solutions with final stress values of 0.11101 (animal- and mycoparasites), 0.09244 (ectomycorrhizal fungi), 0.05238 (lichens and lichenicolous fungi), 0.12336 (plant pathogens) and 0.07267 (saprotrophs), with final instability values less than 0.00001. The NMDS plots revealed strong structuring in all functional groups with tundra type being the most influential variable (table 2 and figure 1). Similarly, Venn diagrams indicated that a substantial fraction of OTUs were unique to a tundra type (electronic supplementary material, figure S1). Warming had strong effect on the fungal community in the moist tundra, where community composition was significantly different between treatment and control in all functional groups. However, in the dry tundra, only plant pathogens showed significant treatment effect on composition (table 2).

**Table 2.** Effects of tundra type and warming on community composition of functional groups of fungi as calculated using multi-response permutation procedure. Significant *p*-values are indicated in italics.

functional groups	tundra type		warming in dry tundra		warming in moist tundra	
	effect (*A*)	*p*	effect (*A*)	*p*	effect (*A*)	*p*
ectomycorrhizal	0.15236	*<0.00001*	0.0219	0.07663	0.10865	*0.00197*
animal parasites and mycoparasites	0.1153	*0.00002*	0.01459	0.69563	0.14281	*0.00196*
lichens and lichenicolous fungi	0.21142	*<0.00001*	0.00677	0.28502	0.15166	*0.01258*
plant pathogens	0.18262	*<0.00001*	0.04895	*0.03357*	0.09515	*0.00308*
saprotrophs	0.19335	*<0.00001*	0.01331	0.16814	0.08925	*0.00389*

### (c) Abundance at the species-level

**Figure 2.**
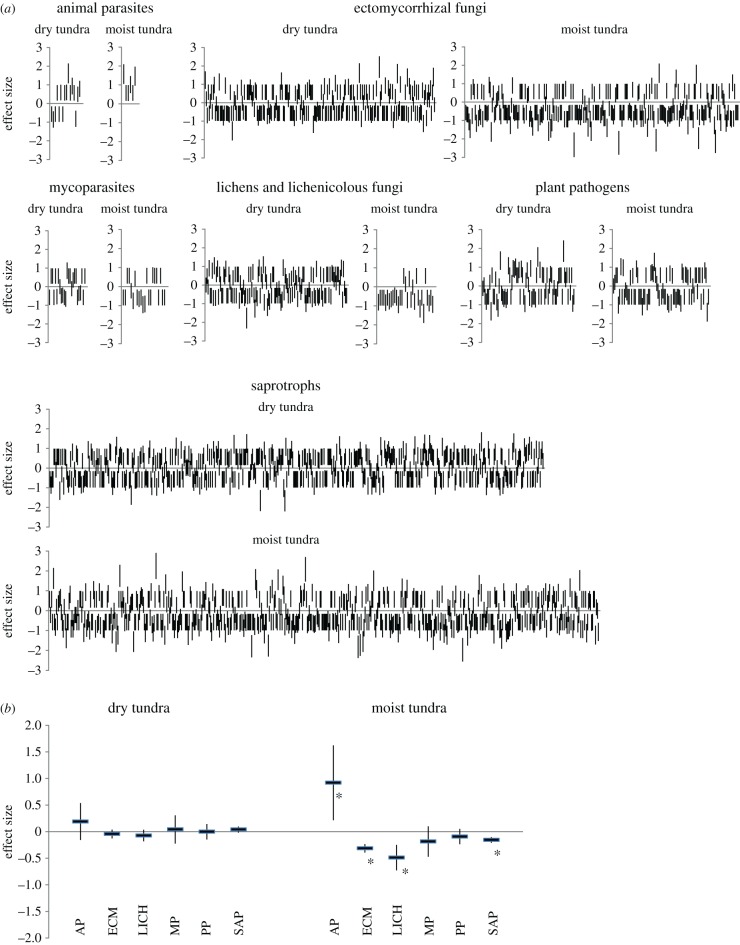
(*a*) Responses of individual OTUs in the functional groups to warming. Each vertical line represents the effect of warming on mean DNA sequence read count with variance for a fungal OTU. Positive and negative effects indicate increased and decreased abundance in the warmed plots, respectively. (*b*) Summarized responses of functional groups of arctic fungi to warming. The values represent the mean effect size and 95% confidence interval from meta-analyses of all OTUs in the functional group in question. Abbreviations: AP, animal parasites; ECM, ectomycorrhizal fungi; LICH, lichens and lichenicolous fungi; MP, mycoparasites; PP, plant pathogens; SAP, saprotrophs. (Online version in colour.)

Sequence read counts of most OTUs differed between the control and treatment as indicated by non-zero effect values and their variance intervals (figure 2*a*). Meta-analyses of trends of the individual OTUs per functional group indicated significant changes only in the moist tundra, where we observed significant decline in ectomycorrhizal, lichenized and saprotrophic fungi, as well as significant increase in animal pathogens, while mycoparasites and plant pathogens showed non-significant decline (figure 2*b*).

## 4. Discussion

Tundra type greatly affected fungal communities, with shifts in composition and OTU abundance in response to warming being stronger in the moist as opposed to the dry tundra. Because most fungal symbiotic plants occur in both vegetation types, the profound fungal compositional differences between moist and dry tundra are probably caused by well-known differences in fundamental abiotic attributes, such as snow cover, active layer depth, soil moisture, nutrients and temperature [7]. The accumulating evidence in this and other studies [8,19] suggests that warming responses of fungal and plant communities probably are predicated on soil water conditions and resulting differences in productivity among tundra types.

Changes in communities of arctic fungal functional groups have been scarcely documented, except in ectomycorrhizal fungi [10]. The compositional differences between the warmed and control plots in all functional groups indicate that even in groups without major changes in richness, the turnover is substantial. Although such compositional shifts are particularly evident in the moist tundra, parasites, ectomycorrhizal fungi and pathogens also display clearly visible warming-induced changes in the dry tundra (figure 1 and table 2).

The high proportion of OTUs with marked changes in abundance is striking (figure 2*a*). Even in the dry tundra, where the overall effect size of warming was not significant, most OTUs showed a clear trend, with only a small fraction of OTUs seemingly unaffected by warming. This indicates that response to warming probably is species-specific within these broad ecological groups.

Overall trends were more profound in the moist tundra, where significant changes were observed in most functional groups (figure 2*b*). The only increase was in animal parasites, which is in agreement with observed warming-induced increases in insect abundance [20]. All OTUs of animal parasites in the moist tundra were positively affected by warming and even in the dry tundra this group showed the largest, although not significant, increase. Abundance decrease in ectomycorrhizal fungi may have functional implications and the fact that several ectomycorrhizal fungi showed positive response to warming, while most were negatively affected, indicates substantial shift in the community. The strong decrease in lichen abundance underlines their decrease in cover due to increased shading by shrubs in the warmed moist tundra [8]. In the dry tundra, where shading is minimal, several lichens benefited from warming (figure 2*a*). The decrease in saprotrophs is surprising in the light of non-significant changes in richness (above) and previous findings on warming-induced increase in litter accumulation [8] and in microbial decomposition rates [21]. However, distinct species-specific responses to warming were revealed also in saprotrophic taxa.

In this paper, we provide evidence that long-term experimental summer warming has profound effects on community composition and abundance of functional groups of arctic fungi. We also underline that, while there are similarities within functional groups, changes in occurrence and abundance in response to warming tend to be species-specific, and may be masked when communities are compared at higher taxonomic levels. Therefore, we recommend that studies of arctic fungal communities (for example, their roles in nutrient cycling) take into account species-level differences. Finally, we advocate the integration of taxonomic and functional data into climatic models to better understand the influence of climate on soil microbial community structure and function and their contributions to climate-linked processes.

## Supplementary Material

STable 1

## Supplementary Material

SFigure 1

## Data accessibility

All molecular data are publicly available in Dryad: http://dx.doi.org/10.5061/dryad.2fc32 [22].
