# Supplementary material for: Changes in composition and abundance of functional groups of arctic fungi in response to long-term summer warming
Source: Biol Lett. 2016 Nov;12(11):20160503. doi: 10.1098/rsbl.2016.0503 (PMC5134034; doi:10.1098/rsbl.2016.0503)
Supplement: STable 1 [file rsbl20160503supp1.docx]

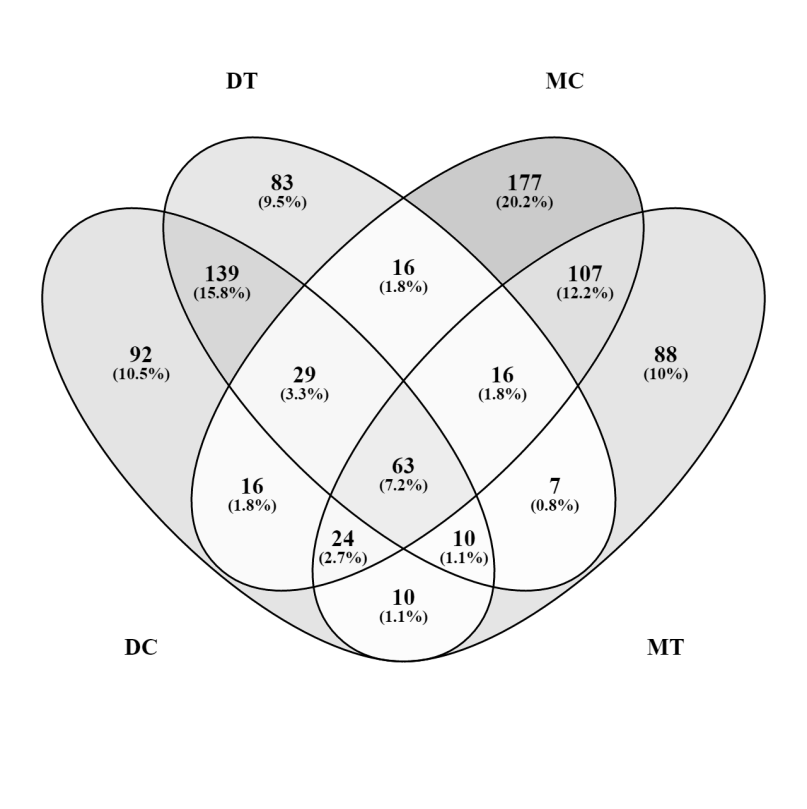

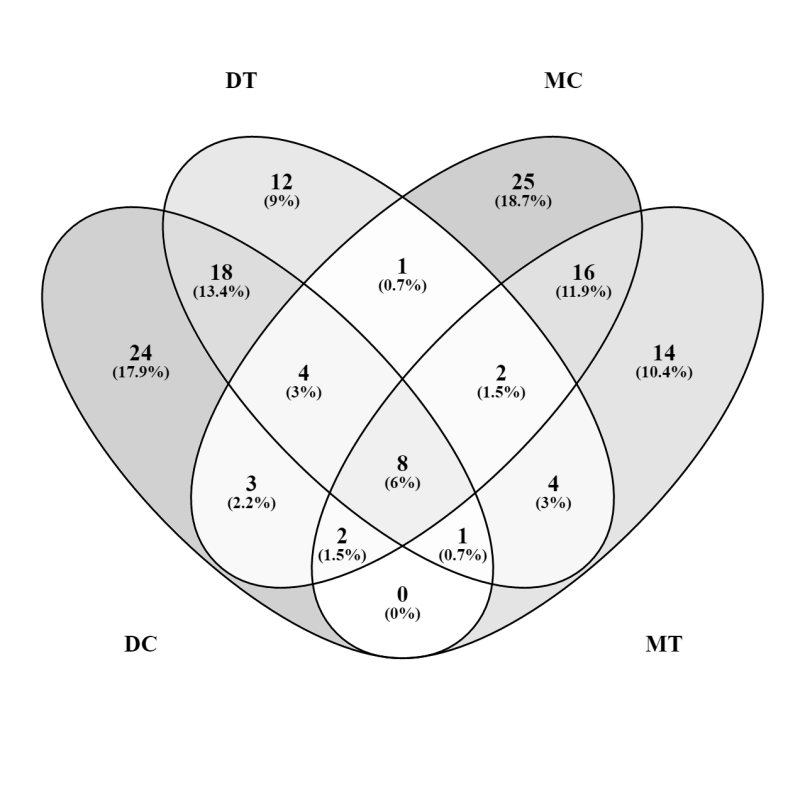


**Saprotrophic**

**Plant pathogenic**


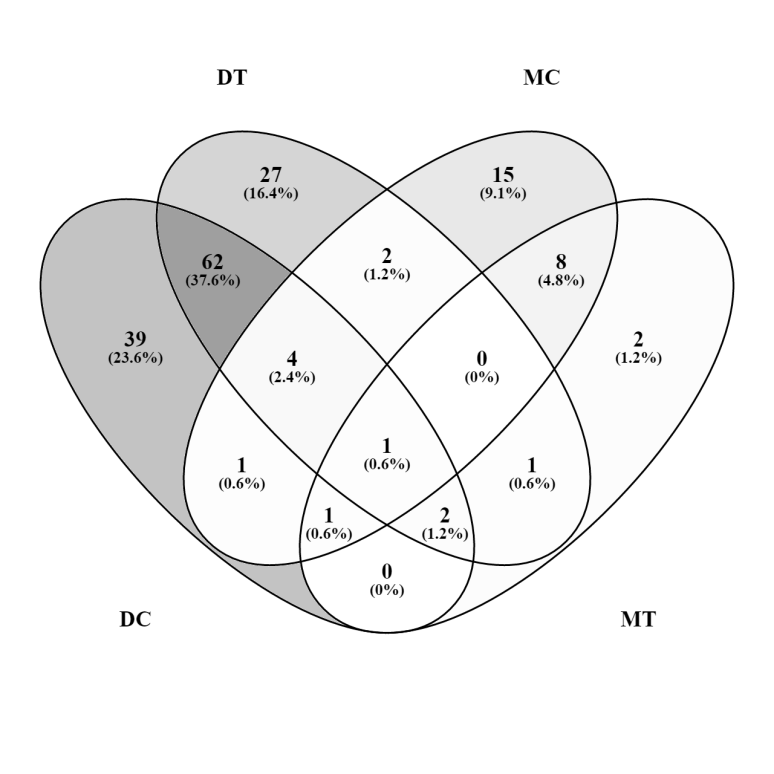

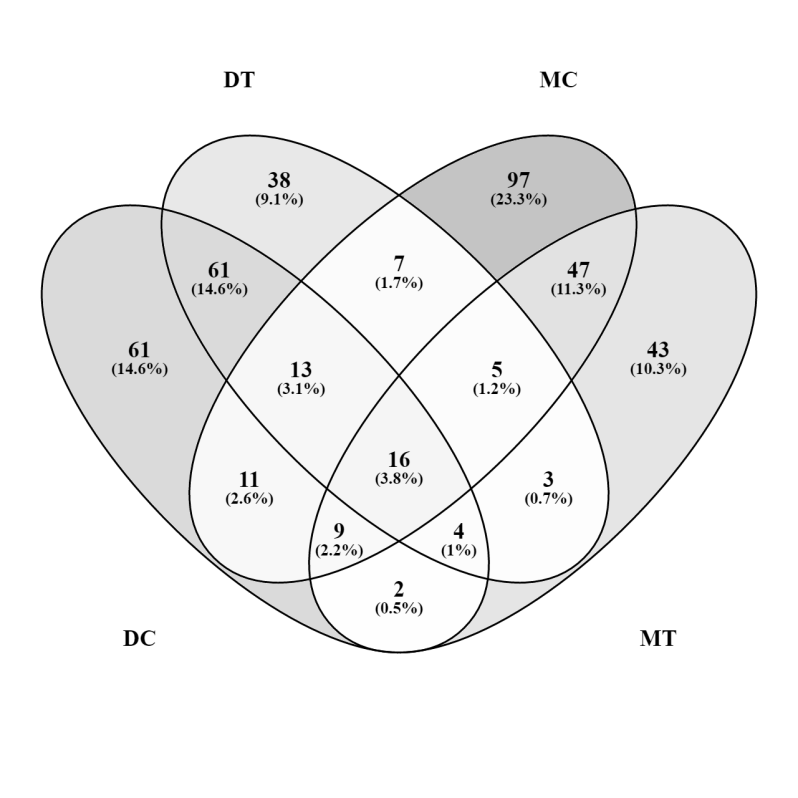


**Ectomycorrhizal**

**Lichenized**


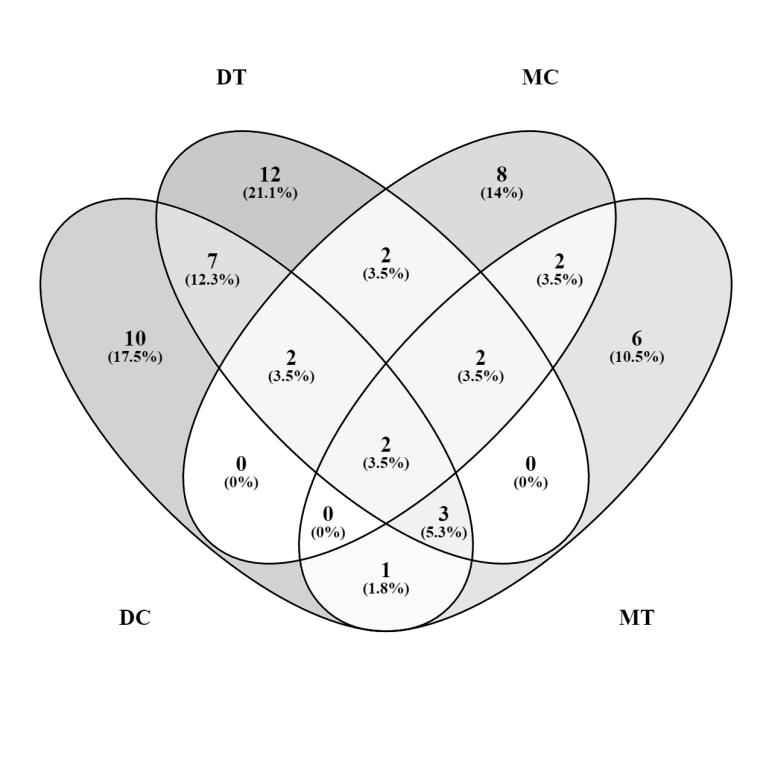


Supplementary Information for the manuscript:

*Changes in composition and abundance of functional groups of arctic fungi in response to long-term summer warming*

by József Geml, Tatiana A. Semenova,, Luis N. Morgado, Jeffrey M. Welker

SFigure 1. Venn diagrams of the five major functional groups of fungi showing the distribution of OTUs among tundra and treatment types. M = moist tundra, D = dry tundra, C = control, T = warming.

**Animal parasites**
